# The Fold-in, Fold-out Design for DCE Choice Tasks: Application to Burden of Disease

**DOI:** 10.1177/0272989X19849461

**Published:** 2019-05-29

**Authors:** Lucas M. A. Goossens, Marcel F. Jonker, Maureen P. M. H. Rutten-van Mölken, Melinde R. S. Boland, Annerika H. M. Slok, Philippe L. Salomé, Onno C. P. van Schayck, Johannes C. C. M. in ‘t Veen, Elly A. Stolk, Bas Donkers, on behalf of the research team that developed the ABC tool

**Affiliations:** Erasmus School of Health Policy and Management & Institute for Medical Technology Assessment, Erasmus University Rotterdam, Rotterdam, the Netherlands; Erasmus Choice Modelling Centre, Erasmus University Rotterdam, Rotterdam, the Netherlands; Erasmus School of Health Policy and Management & Institute for Medical Technology Assessment, Erasmus University Rotterdam, Rotterdam, the Netherlands; Erasmus Choice Modelling Centre, Erasmus University Rotterdam, Rotterdam, the Netherlands; Erasmus School of Health Policy and Management & Institute for Medical Technology Assessment, Erasmus University Rotterdam, Rotterdam, the Netherlands; Erasmus Choice Modelling Centre, Erasmus University Rotterdam, Rotterdam, the Netherlands; Erasmus School of Health Policy and Management & Institute for Medical Technology Assessment, Erasmus University Rotterdam, Rotterdam, the Netherlands; Erasmus Choice Modelling Centre, Erasmus University Rotterdam, Rotterdam, the Netherlands; CAPHRI Care and Public Health Research Institute, Maastricht University, Maastricht, the Netherlands; UNICUM Huisartsenzorg, Bilthoven, the Netherlands; CAPHRI Care and Public Health Research Institute, Maastricht University, Maastricht, the Netherlands; Department of Pulmonology, Franciscus Gasthuis en Vlietland, Rotterdam, the Netherlands; Erasmus School of Health Policy and Management & Institute for Medical Technology Assessment, Erasmus University Rotterdam, Rotterdam, the Netherlands; Erasmus Choice Modelling Centre, Erasmus University Rotterdam, Rotterdam, the Netherlands; EuroQol Foundation, Rotterdam, the Netherlands; Erasmus Choice Modelling Centre, Erasmus University Rotterdam, Rotterdam, the Netherlands; Erasmus School of Economics, Erasmus University Rotterdam, Rotterdam, the Netherlands

**Keywords:** Discrete choice experiments, Task complexity, Preference measurement, COPD, Burden of disease

## Abstract

**Background** In discrete-choice experiments (DCEs), choice alternatives are described by attributes. The importance of each attribute can be quantified by analyzing respondents’ choices. Estimates are valid only if alternatives are defined comprehensively, but choice tasks can become too difficult for respondents if too many attributes are included. Several solutions for this dilemma have been proposed, but these have practical or theoretical drawbacks and cannot be applied in all settings. The objective of the current article is to demonstrate an alternative solution, the fold-in, fold-out approach (FiFo). We use a motivating example, the ABC Index for burden of disease in chronic obstructive pulmonary disease (COPD). **Methods** Under FiFo, all attributes are part of all choice sets, but they are grouped into domains. These are either folded in (all attributes have the same level) or folded out (levels may differ). FiFo was applied to the valuation of the ABC Index, which included 15 attributes. The data were analyzed in Bayesian mixed logit regression, with additional parameters to account for increased complexity in folded-out questionnaires and potential differences in weight due to the folding status of domains. As a comparison, a model without the additional parameters was estimated. **Results** Folding out domains led to increased choice complexity for respondents. It also gave domains more weight than when it was folded in. The more complex regression model had a better fit to the data than the simpler model. Not accounting for choice complexity in the models resulted in a substantially different ABC Index. **Conclusion** Using a combination of folded-in and folded-out attributes is a feasible approach for conducting DCEs with many attributes.

Discrete-choice experiments (DCEs) are globally recognized as a valuable instrument to measure preferences of respondents in marketing, environmental economics, transportation, and increasingly health sciences and health economics.^1^ They have been used to address a broad range of questions, including assessing experiences of patients,^2^ tradeoffs between health outcomes and experience factors,^3^ priority setting,^4^ identifying groups with different preferences,^5^ and estimating utility weights for quality-of-life questionnaires.^6^

A DCE offers respondents a series of choices between 2 or more alternatives. These alternatives are described on the basis of characteristics (i.e., attributes), which can take different levels. For instance, attributes of a medical treatment might be the risk of a serious adverse event (with possible levels: 1%, 10%, and 20%) or copayment (levels: nothing, €100, and €200). In each DCE question, 2 or more alternatives are presented, from which respondents have to choose the most appealing one. Attribute levels vary across the alternatives, so one alternative may be more attractive in terms of risk, whereas the other alternative may be more attractive in terms of copayment. Using statistical choice models, researchers can infer from these choices how important the various attributes are for the respondents. The coefficients of these models quantify the relative importance of the various attribute levels, and it is possible to sum up the coefficients to compute utilities of alternatives.

A challenge arises in the application of DCEs when there are many attributes involved in a decision, because choices become more difficult or even infeasible for respondents if the number of attributes increases.^7,8^ Many researchers seem to put a large weight on this argument and aim to comprehensively describe the decision problem using no more than 6 or 7 attributes.^1^ Unfortunately, leaving out attributes is not always desirable.

Several strategies have been proposed for conducting a DCE with a large number of attributes.^9–11^ However, they require additional assumptions that might not always hold. The first strategy is called “partial profiling”^12^ or “blocked attribute design.”^11^ In this approach, respondents get to see only a selection of attributes (a partial profile or a block) at a time. The omitted attributes are assumed to be equal across choice alternatives. With different attributes being presented in different choices, the same respondent gets to see different selections of attributes, so the full range of attributes is covered. The analysis is usually based on the pooled data from all blocks. This assumes that the preferences of respondents for the presented attribute levels are independent of omitted attributes. When Witt et al.^11^ analyzed the data from each block separately, they noted that the coefficients for the common attributes differed across blocks. This suggested that marginal utility and the marginal rate of substitution can be sensitive to the inclusion of other attributes.

In the second strategy, the hierarchical information integration (HII) method,^9,10^ several individual attributes are grouped into overarching constructs. These constructs can then be used in choice tasks, replacing the original attributes. The relative weight of attributes belonging to a construct is investigated in a separate rating task (“subexperiment”). This reduces the number of attributes in the final choice tasks (“bridging experiment”) and decreases the burden on respondents.

HII assumes that respondents would already mentally group similar attributes into constructs when they make decisions and are able to meaningfully and sensibly attach one summarizing value to a group of attributes, which also validly reflects the value of the underlying attributes. This is not always evident, and the obtained value may in fact become dependent on the used labels for the overarching constructs. For this reason, the analysis usually contains a test of construct validity.^10^ However, such a test shows whether attributes contribute to the level of the construct. It does not show whether the value of the construct in the DCE is accurately perceived by respondents. Furthermore, the test is often performed only after the choice data have been collected. Although HII was developed decades ago, there have been few applications of it in health care so far.^13,14^

The objective of the current article is to present an alternative solution to the problem of large numbers of attributes, the fold-in, fold-out approach (FiFo). We developed this approach in the context of a study in which we aimed to elicit preference values for the 15 items of the Assessment of the Burden of Chronic Obstructive Pulmonary Disease (ABC) tool to create the ABC Index. Under FiFo, attributes are categorized into domains, but in contrast to HII, they are not replaced by them. Creating such overarching constructs also would not have made much sense in our context. Under FiFo, respondents can be presented domains that are either folded in or folded out. When a domain is folded in, all its attributes are still shown but have the same level. When a domain is folded out, the levels may differ across attributes. The scores of the ABC Index were published elsewhere.^15^ In the current article, we focus on demonstrating the innovative methodology (i.e., the FiFo design of the questionnaire and the analysis of the choice data).

## Methods

### Context: The ABC Index

The ABC tool was recently developed to support shared decision making by patients and health care providers.^16^ It includes the ABC questionnaire about the experienced burden of chronic obstructive pulmonary disease (COPD) (see Figure A1 in the online appendix), and several objective indicators, such as smoking behavior and a colored balloon diagram, to visualize patients’ scores. These scores are translated into a treatment advice to be discussed with the patients. The resulting individual care plan includes personal treatment goals framed in the patient’s own words. In an 18-month cluster randomized controlled trial (RCT), this tool was found to be effective in improving disease-specific quality of life.^17^

As an addition to the tool, the ABC Index was developed. The index aggregates the scores of the items of the ABC questionnaire into a total score for the experienced burden of disease, based on the importance of each item for patients. The ABC Index is the first preference-based measure of burden of disease in COPD. It can be used to monitor a patient’s overall improvement or deterioration, describe populations of patients in terms of experienced burden of disease, and assist in contracting between health care insurers and providers. It was shown to be predictive of health care consumption and costs.^15^

### Discrete-Choice Experiment

In the development of the ABC Index, the importance of each item of the ABC tool was assessed in a DCE, which consisted of a series of pairwise choice tasks. In each choice task, the health states of 2 COPD patients were described. Respondents were asked to indicate which of them they considered to be in worse health. They were presented with 14 choice tasks, each describing 2 COPD patients in different health states. The first choice task served as a control question to test the respondent’s comprehension of the task. It was not included in the analysis.

### Choice of Attributes and Levels

Each health state was described by 15 attributes from the ABC tool, which can be divided into 3 domains plus 2 separate attributes: a respiratory symptoms domain (with 4 attributes: shortness of breath at rest, shortness of breath during physical activity, coughing, and sputum production), a limitations domain (4 attributes: limitations in strenuous physical activities, limitations in moderate physical activities, limitations in daily activities, and limitations in social activities), a mental problems domain (5 attributes: feeling depressed, fearing that breathing gets worse, worrying, listlessness, and tense feeling), a fatigue attribute, and an exacerbations attribute.

Each attribute had 3 possible levels. The original ABC Questionnaire has 7 answer categories for most questions. However, 7 levels per attribute in a DCE are generally considered too many. The multitude of possibilities and the subtle differences between them would make it more difficult for respondents to distinguish the severity of health states. In addition, it would require the estimation of many coefficients in the statistical analysis. For these reasons, the number of levels of each attribute in the DCE was limited to 3: 1) never or hardly ever, 2) regularly, and 3) most times for the attributes in the respiratory symptoms domain, the mental problems domain, and the fatigue attribute; 1) hardly or not at all, 2) moderately, and 3) severely for the attributes in the limitations domain; and 0, 1, and 2 exacerbations per year for the exacerbation attribute.

#### Fold-in, fold-out design

Without further adjustments, the cognitive burden of the DCE to respondents would be very high. To indicate which of the 2 COPD patients had a worse health status, they would have to make tradeoffs between 15 attributes.

The new FiFo design was developed to ease this burden. Unlike the HII design, FiFo does not create a new overarching construct that fully replaces the attributes in that construct. Instead, it shows all attributes in every choice task, but some domains are “folded in” while others are “folded out.”

**Figure 1:**
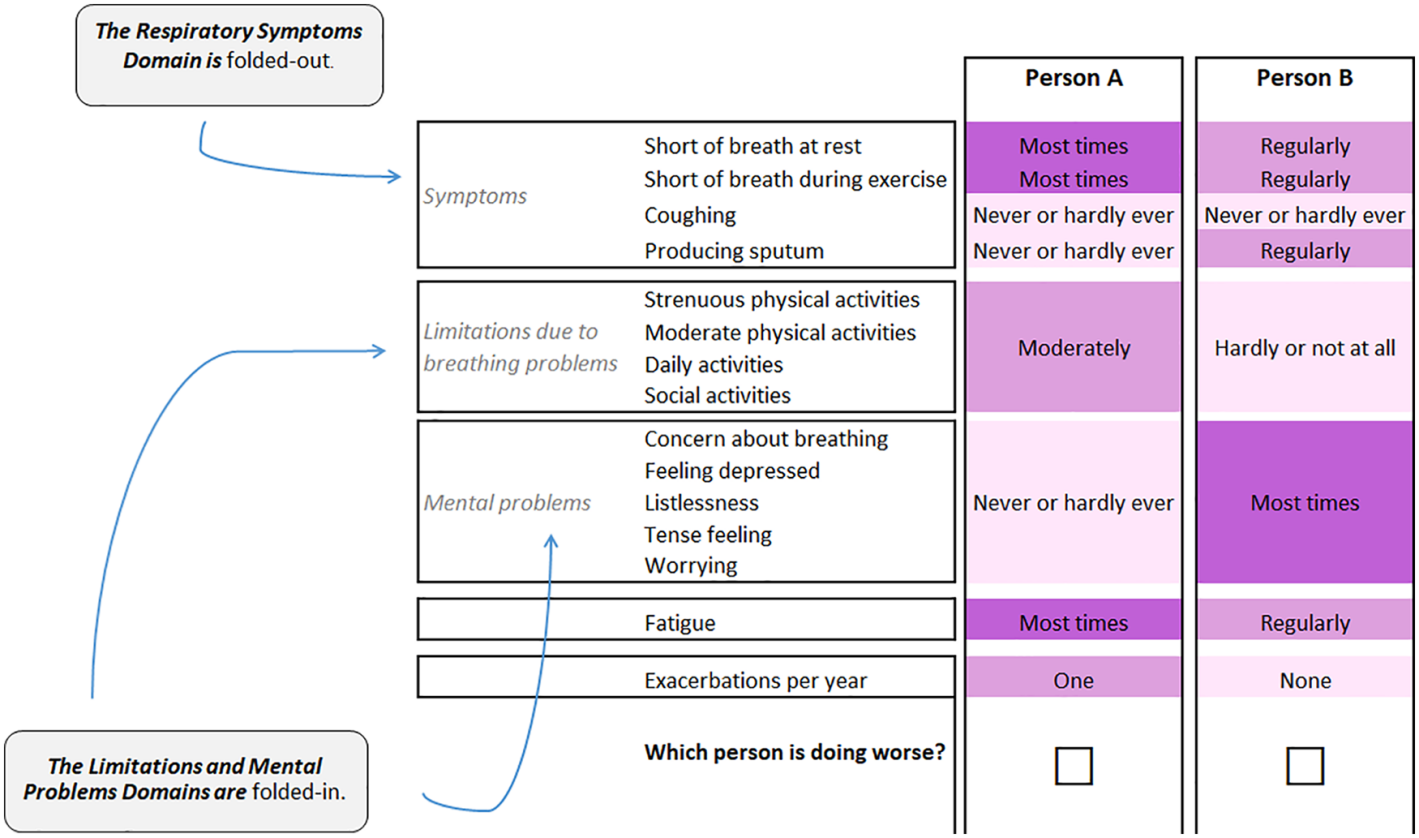
Example of a choice set with a fold-in, fold-out design. Attribute levels are color-coded, with darker shades for the more severe levels. The color-coding system was optimized for individuals with colorblindness.

When a domain is folded in, all attributes in this domain are forced to be at the same level. When a domain is folded out, the levels of each attribute in a domain are presented separately. In the example of a choice task in Figure 1, the limitations domain and the mental problems domain are folded in. Person A is “moderately” limited in all attributes of the limitations domain, while person B is “hardly or not at all” limited. All mental problems occurred “never or hardly ever” in person A and “regularly” in person B. In contrast with these 2 domains, the respiratory symptoms domain is folded out (i.e., the levels differ across the attributes within the domain).

In every choice task, 2 or 3 domains were folded in and 1 was folded out. To facilitate comparison, domains would be either folded in or out in both alternatives.

The attribute levels were color-coded, with darker shades for the more severe levels. This color-coding system was specifically optimized to signal differences in the attribute levels for individuals with red-green colorblindness (the most prevalent form of colorblindness) while keeping the text readable for respondents who have other forms of colorblindness^18^ and was reported to reduce task complexity for respondents.^19^

Figures A2 and A3 in the online appendix present choice tasks in the formats of partial profiling and HII, respectively, in the context of the ABC Index. These formats were not used in this study. They serve as a clarification of these alternative approaches and to show the contrast with the FiFo format.

#### Efficient factorial design

Each respondent got 14 choice tasks: 6 choice tasks with all domains folded in and 8 choice tasks with one of the domains folded out.

With 15 attributes and 3 possible levels per attribute, many possible choice tasks could be formed. The experimental design was optimized using Ngene (ChoiceMetrics Ptd Ltd, Sydney, Australia) using a D-efficiency design optimization criterion with fixed priors, given the conditions of the FiFo design. This was done separately for each DCE question type: that is, questions with all domains folded in and with the symptoms, limitations, or mental problems domain folded out, respectively. For each question type, 4 (folded-out) or 6 (folded-in) blocks of questions were developed. Finally, questionnaires were assembled by combining the control question and 1 block of 5 questions with all domains folded in with a block of 8 questions from a type with a domain folded out. Combinations were made based on attribute level balance. All blocks with folded-out domains were used once, while the blocks with only folded-in domains were used in 2 combinations. Finally, all choice sets were copied and options A and B were switched in the second version. This led to a total of 24 different DCE questionnaires, which were randomly assigned to respondents.

To further optimize the efficiency of the design aimed at measuring preferences of the general public, several additional design optimizations were implemented using prior preferences obtained from an analysis of the responses of preceding respondents. This was done after half of the patients had answered the questionnaire; it was repeated 3 times for the members from the general public. This made new designs increasingly more efficient based on the analysis of earlier respondents. This is best practice in DCE research.

The questionnaire was pilot-tested in a sample of patients in Franciscus Gasthuis hospital in Rotterdam (*n* = 10). After the think-aloud interviews, the final layout and wording of the survey was implemented (see Figure 1).

### Respondents

Two groups of respondents took part in the study. The first group consisted of patients who participated in the cluster RCT in which the ABC tool was tested.^17^ They were recruited in 56 health care centers across the Netherlands (39 primary care, 17 hospital care) between March 2013 and October 2013 and interviewed between January and June 2015. All interviews were held after the patients had completed the trial. The second group consisted of a nationally representative sample (in terms of age, sex, and education level) of the general public, who were approached through a survey sample provider.

The questionnaires were administered in telephone interviews by trained researchers from Erasmus University Rotterdam using a (semi)structured interview protocol. Questionnaires were sent to respondents by mail or email before the interview took place, so respondents had the choice questions in front of them during the phone call.

Interviewers started by explaining to patients that the experiment was only about their opinions and that it would not have any consequences for their individual treatments. The interviews with members of the general public started with an explanation of the disease COPD and their possible experience with it in their family or circle of friends. Both groups were then asked to complete 2 simplified tasks and 1 realistic practice choice task before the actual DCE questionnaire started.

At the end of the interview, 3 debriefing questions were asked about the difficulty of the questionnaire. Participating respondents received a €20 reward after completing the interview.

### Analysis

A Bayesian mixed logit regression model was used to analyze the choices. DCE regression models assume that choices are determined by the utility that respondents attach to the alternatives. This utility has an observable component, which is described by attribute levels and regression parameters, and an unobservable part, which is captured by the error term.

Hence, utility *U* of alternative *i* for respondent *n* can be expressed as

$$U_{\mathrm{ni}}=\beta \prime X_{\mathrm{ni}}+\varepsilon_{\mathrm{ni}},$$

where $X_{i}$ is a vector of attribute levels that describe alternative *i*, and $\beta_{n}$ is a vector of parameters for the effects of these attribute levels on the utility of this alternative for the *n*th respondent. The error term $\varepsilon_{\mathrm{ni}}$ has a standard type I extreme value distribution.

Mixed logit allows the regression coefficients β to vary across respondents, thereby taking into account the systematic differences in preferences across respondents. The general expression of the mixed logit probability for alternative *i* in choice task *j* being chosen by respondent *n* is

$$P_{\mathrm{ni}}=\int \left(\frac{e^{\beta \prime x_{\mathrm{ni}}}}{\sum_{j}e^{\beta^{\prime x_{\mathrm{nj}}}}}\right)f\left(\beta |b,W\right)d\beta ,$$

where *P* denotes the probability of an alternative being chosen, and f(β|b,W) describes a distribution of parameter values across respondents. In our case, it was a normal density function with mean *b* and variance *W*.

Given the fold-in, fold-out-presentation of the discrete-choice tasks, it seems plausible that it became more difficult for respondents to make choices between 2 alternatives when domains were folded out. This is not adequately incorporated in the standard MIXL model, and hence a modified regression model was developed that explicitly accounted for the fact that the structure of questions was not constant (i.e., in some choice tasks, all domains were folded in, whereas in others, one of the domains was folded out). This was done by varying the scale of the error term for tasks with a folded-out domain.

Accordingly, the expression for utility *U* of alternative *i* for respondent *n* was generalized as

$$U_{\mathrm{ni}}=\beta \prime X_{\mathrm{ni}}+\varepsilon_{\mathrm{ni}}/\sigma .$$

Scale factor σ makes explicit that the scale for utility is arbitrary: the consistency of choice behavior depends on the ratio of parameters to the error term, not on the absolute value of the parameters. Since it is not possible to identify both β and σ at the same time, σ is usually normalized to 1.

To reflect differences in complexity, the scale parameter of the error term was modeled with an additional parameter for choice tasks with a folded-out domain^20,21^:

σ=1+φ.

Identification of this extra φ parameter was ensured by jointly analyzing the data from folded-in and folded-out questionnaires in a single statistical model.

A negative φ parameter would mean that the unobserved, unpredictable component of utility had a relatively larger impact on choices in folded-out questions compared to folded-in questions. This would indicate that respondents’ preferences were less clear and choices were less consistent when one of the domains was folded out.

In a further extension, the weights for the attribute levels were allowed to be different for folded-out domains compared to folded-in domains. This was achieved by extending the utility function with domain-specific λ*-*parameters, which make it possible to vary the weight of the attributes in each domain by their folding-out status:

$$\;U_{\mathrm{ni}}=\left(1+\lambda_{s}*o_{s}\right)*\beta_{s}\prime S_{\mathrm{ni}}+\left(1+\lambda_{l}*o_{l}\right)*\beta_{t}\prime L_{\mathrm{ni}}+\left(1+\lambda_{m}*o_{m}\right)*\beta_{m}\prime M_{\mathrm{ni}}+\beta_{X}\prime X_{\mathrm{ni}}+\varepsilon_{\mathrm{ni}}/\sigma ,$$

where *S_i_, L_i_*, and *M_i_* are vectors of attribute levels in the symptoms, limitations, and mental problems domains, respectively; *X_i_* is a vector of levels of the attributes that are not included in these domains; and *o_s_, o_l_*, and *o_m_* are indicators of the folded-out status of the domains.

The β-parameters represented the weights, as they were perceived by respondents for domains in the folded-in state. Positive λ-parameters indicate that respondents attached more weight to domains that were folded out.

As a comparison, the regression analysis was repeated as a Bayesian mixed logit model without the λ- and φ-parameters. Model fit was assessed using the Watanabe information criterion (WAIC) and the deviance information criterion (DIC).^22,23^ OpenBugs software (open source, www.openbugs.net) was used to fit the regression models and Excel 2013 (Microsoft, Redmond, WA) was used to calculate ABC Index scores. The OpenBugs code is included in the online appendix.

### Calculating the ABC Index Score

The estimation results were used to calculate the ABC Index scores. The worst possible health state was defined to be equivalent to 100 points on the ABC Index scale. A score of 0 points would indicate that a patient was on the best possible level on all attributes (i.e., “not or hardly” limited; “seldom or never” symptoms, mental problems, or fatigue; no exacerbation).

Index scores were calculated by linear interpolation and extrapolation from the 3 coefficients per DCE attribute to the 7-point scale of the original ABC tool. This was complicated by the fact that the β-coefficients in the symptoms, limitations, and mental problems domains represented the weights for attributes as presented in their folded-in state. This made it less than obvious that they should be directly used in the intra- and extrapolation.

On one hand, it could be argued that attributes did not get enough attention, relatively, when they were folded in. This would justify adjusting the coefficients to the folded-out state by multiplying them with an adjustment factor of (1 +λ). On the other hand, it is also plausible that the attention of respondents was exaggeratedly drawn to the folded-out domain after the first 5 choice tasks in the questionnaire, which were all folded in completely. This would be an argument for using the original coefficients. A third option would be to apply a partial adjustment factor (1 + 0.5 * λ). We used all 3 options and calculated 3 versions of the ABC Index, with the partial correction as the base case.

## Results

### Respondents

All 328 COPD patients from the RCT who stayed in the trial for at least 6 months were approached. Most (86%, 283 patients) were willing to participate in the DCE. The duration of the interviews was approximately 30 minutes, and all but 1 patient who started the interview fully completed it. All respondents from the general public completed the interview. Table 1 shows that the characteristics of the respondents from the trial were different from those of the general public. COPD patients were older on average, with less variation in age and less educated. A sizable proportion of the respondents from the general public stated that they were COPD patients themselves.

**Table 1:** Respondents

Characteristic	Patients (*n* = 282)	General Public (*n* = 250)
Age, mean (SD), y	66.7 (8.3)	46.0 (16.7)
Male, %	52	55
COPD, %	100	8.3
GOLD 1, %	12	
GOLD 2, %	54	
GOLD 3, %	31	
GOLD 4, %	3	
Education, %		
Low	48	13
Middle	37	35
High	15	52

COPD, chronic obstructive pulmonary disease; GOLD, GOLD (Global Initiative for Chronic Obstructive Lung Disease) classification 1/2/3/4: mild/moderate/severe/ very severe COPD.

After the choice tasks, most of both respondent groups reported that the task was almost completely or totally clear to them (see Table 2). They agreed that the differences between patients A and B were at least reasonably easy to discern. Members from the general public found this easier than respondents in the patient sample. Making choices was reported to be (very) difficult by 15% of the patients and 13% of the members from the general public.

**Table 2:** Cognitive Debriefing Questions

Question	Patients, %	General Public, %
Was it clear what the task was?		
Not at all	4	0
Not really	1	0
Reasonably	25	6
Almost completely	6	8
Totally	65	86
Was the difference between persons A and B easy to see?		
Not at all	1	0
Not really	3	2
Reasonably	44	20
Almost completely	12	16
Totally	39	63
How easy was it to decide between patients A and B?		
Very difficult	6	4
Difficult	9	9
Doable	50	50
Easy	16	22
Very easy	20	15

### Regression Results

Table 3 shows the results of the regression analyses for patients and the general public, respectively. Two coefficients per item or domain are presented: one for level 1 (moderate problems) and one for level 2 (severe problems). A positive coefficient indicates that respondents considered this level worse than level 0 (no or hardly any problems). A larger coefficient means that the attribute contributes more to the burden of disease.

**Table 3:** Regression Results: Bayesian Mixed Logit Model with Fold-in, Fold-out Parameters

		Patients		General Public	
Attributes and Domains	Level	Coefficient	95% CI	Coefficient	95% CI
Fatigue	Regularly	1.189	0.876 to 1.545	1.023	0.701 to 1.382
	Most times	2.103	1.662 to 2.652	2.377	1.856 to 2.980
Exacerbations	Once a year	0.815	0.516 to 1.162	1.513	1.125 to 1.961
	Twice a year	2.243	1.744 to 2.881	2.642	2.074 to 3.312
Symptoms					
Dyspnea at rest	Regularly	0.404	−0.021 to 0.893	0.294	−0.167 to 0.816
	Most times	1.496	0.872 to 2.302	0.824	0.198 to 1.509
Dyspnea during exercise	Regularly	0.330	−0.211 to 0.873	0.503	−0.064 to 1.087
	Most times	0.470	−0.065 to 1.021	1.195	0.591 to 1.868
Coughing	Regularly	0.172	−0.297 to 0.623	0.810	0.373 to 1.310
	Most times	0.465	−0.025 to 0.959	1.034	0.542 to 1.577
Sputum	Regularly	0.766	0.395 to 1.221	0.444	0.063 to 0.865
	Most times	0.528	0.057 to 0.993	1.252	0.766 to 1.836
Limitations					
Strenuous physical activities	Moderately	0.544	−0.049 to 1.107	0.578	−0.345 to 1.403
	Severely	0.692	0.033 to 1.322	0.921	−0.058 to 1.850
Moderate physical activities	Moderately	0.763	0.239 to 1.333	0.858	0.100 to 1.832
	Severely	1.287	0.629 to 2.039	1.692	0.722 to 2.997
Daily activities	Moderately	0.320	0.005 to 0.656	0.344	−0.085 to 0.816
	Severely	1.028	0.540 to 1.589	1.099	0.364 to 1.866
Social activities	Moderately	0.245	−0.073 to 0.588	0.563	0.125 to 1.079
	Severely	0.943	0.440 to 1.483	1.392	0.666 to 2.168
Mental problems					
Fearing breathing problems	Regularly	0.655	0.255 to 1.089	0.706	0.247 to 1.208
	Most times	1.461	0.886 to 2.172	1.587	0.898 to 2.322
Feeling depressed	Regularly	0.057	−0.378 to 0.480	1.027	0.551 to 1.561
	Most times	0.729	0.287 to 1.199	1.610	1.100 to 2.207
Listlessness	Regularly	0.440	0.008 to 0.872	0.320	−0.149 to 0.818
	Most times	0.512	0.060 to 0.973	0.967	0.454 to 1.542
Tense feeling	Regularly	0.751	0.338 to 1.233	0.177	−0.296 to 0.642
	Most times	0.885	0.312 to 1.526	1.024	0.381 to 1.728
Worrying	Regularly	0.381	−0.077 to 0.828	0.722	0.273 to 1.215
	Most times	0.797	0.217 to 1.388	0.542	−0.105 to 1.227
Adjustment parameters					
Lambda symptoms		0.490	0.036 to 0.960	0.481	0.128 to 0.864
Lambda limitations		1.254	0.754 to 1.817	0.542	0.178 to 0.940
Lambda mental problems		0.387	0.047 to 0.764	0.392	0.194 to 0.708
Phi (complexity parameter)		−0.531	−0.654 to −0.387	−0.528	0.105 to −0.376

CI, credible interval around the estimate of the coefficient.

All coefficients for the items and domains had the expected positive sign, with larger coefficients at the higher levels. Patients and members of the general public had similar but not equal preferences. Both attached much weight to the number of exacerbations, fatigue, limitations at moderate physical activities, and concern/fear of breathing problems getting worse.

The positive λ-parameters indicate that both groups of respondents attached less weight to domains when they were folded in and more when they were folded out. The negative φ-parameter indicated that respondents’ preferences were less clear and choices were less consistent when one of the domains was folded out compared to questions with only folded-in domains.

The goodness of fit was better for the models with additional parameters than for the standard models. The WAIC and DIC for the former were lower (see Table 4) in both groups of respondents.

**Table 4:** Goodness of Fit: Comparison of Results per Statistical Method

Variable	Standard Mixed Logit Models	Bayesian Mixed Logit with Additional Parameters
Patients		
WAIC	4165	4239
DIC	3760	3817
General public		
WAIC	3790	3846
DIC	3427	3445

DIC, deviance information criterion; WAIC, Watanabe information criterion.

### ABC Index Scores

The choice of the analysis method and the choice of the adjustment factor have a substantial impact on the resulting index scores. The scores for the worst possible levels are shown in Table 5.

**Table 5:** ABC Index Scores for the Worst Possible Levels: Comparison of Results, per Statistical Model and Choice of Adjustment Factors

	Bayesian Mixed Logit Regression			
	Without Additional Parameters	With Additional Parameters		
Variable		No Adjustment^a^	Full Adjustment^a^	Partial Adjustment^a^
Fatigue	14	14	9	11
Symptoms				
Dyspnea at rest	11	11	10	10
Dyspnea during exercise	3	3	3	3
Coughing	2	3	3	3
Sputum	2	2	2	3
Mental problems				
Fearing breathing problems	10	10	9	9
Feeling depressed	5	6	5	5
Listlessness	2	3	2	2
Tense feeling	5	5	5	5
Worrying	6	5	5	5
Limitations				
Strenuous physical activities	0	4	6	5
Moderate physical activities	14	8	12	11
Daily activities	8	7	11	9
Social activities	6	7	10	9
Exacerbations	12	12	8	10

a. Coefficients were not, fully, or partially adjusted to folded-out status by multiplying them with 1, 1 +λ, or (1 +0.5 * λ), respectively.

In the standard Bayesian mixed logit model without additional parameters, the highest scores were for limitations at moderate physical activities, fatigue, exacerbations, dyspnea at rest, and fear of breathing problems getting worse. The lowest possible score was assigned to limitations in strenuous physical activities, which would suggest that patients care less about these limitations.

Besides showing a better fit to the data, the results of the extended models with additional parameters showed clear differences with those of the conventional model. First, the scores in the limitations domain were markedly different. Especially, limitations at strenuous physical activities had a greater impact on utility than in the conventional model, whereas limitations at moderate physical activities had a smaller impact on utility. Second, scores for fatigue and exacerbations were lower if the adjustment was applied at least partially. With regard to the symptoms and mental problems domains, all models led to more or less similar scores.

Comparing the 3 approaches with additional parameters to correct for differences between the fold-in and fold-out domains, it becomes clear that more adjustment led to lower scores for exacerbations and fatigue and higher scores for limitations in all activities.

## Discussion

This article presented the first application of the FiFo approach in DCEs with many attributes. FiFo reduces the cognitive burden on respondents, while presenting them with all attributes in all choice tasks. This approach is an alternative to hierarchical information integration and partial profiling, which were developed earlier to help DCE respondents cope with a multitude of attributes. FiFo is a feasible alternative because we have shown that both patients and the general public were able to complete the choice tasks, and the results in both groups of respondents were very plausible, without excluding any patient from the analyses.

FiFo requires an extended analysis method. Applying the extended regression model instead of a conventional analysis led to markedly different estimates of the preference weights for the ABC Index. Furthermore, it confirmed that respondents experience a heavier cognitive burden when they are confronted with more variety in attributes and attribute levels. This was indicated by the complexity parameter in the regression results in both samples. The negative value shows that the utility scale for folded-out choice tasks is smaller than the scale for folded-in choice tasks. In other words, the coefficients for a DCE with only folded-out questions would have been considerably smaller because respondents would be less certain of their choices. This does not mean that folding in or folding out per se has an effect on the validity of the estimated weights for the ABC Index, only on the reliability. The ABC Index is based on a linear extrapolation of the regression results, so only the relative differences between coefficients matter, not the absolute differences.

Our study also found that respondents attached more weight to attributes when their domains were folded out or, equivalently, less weight to attributes in domains that were folded in. That result might have been predicted by support theory.^24,25^ According to support theory, respondents judge the probability of an event to be higher when the explicitness of the event description increases and more details are given about possible versions of the event. Support theory was developed in a very different context. Our study is not about probabilities. However, the analogy is in the different reactions of respondents to options that are described more or less extensively.

We put forward 2 reasons why respondents attached more weight to attributes in folded-out domains. First, these domains could have more salience because of the way in which they are presented. Alternatively, respondents might react to a change in the format of the choice task questions where all domains folded in were followed by questions with 1 folded-out domain. It is also plausible that both mechanisms work at the same time. Support theory supports the first explanation, without ruling out the second one. This suggests that respondents focus less on folded-in domains than on folded-out domains, regardless of the contrast with earlier choice sets. That would mean that the λ-parameters, which model the folding status of domains, should be incorporated in the transformation of coefficients to index scores, at least in part. Further research could shed more light on this element of the choice behavior of respondents.

Several potential solutions are now available for the problem of attribute overload. FiFo has some potential advantages compared to HII and partial profiling. It is conceptually simpler for respondents than HII. Furthermore, it does not require the creation of overarching constructs, which consist of underlying attributes. This limits the applicability of HII. In our case, using overarching constructs would not have been sensible. However, Oppewal et al.^10^ have shown examples where HII was useful.

In contrast with partial profiling, FiFo presents respondents with all attributes in all choice tasks, which increases the consistency and validity of regression estimates. It also takes into account differences in utility scales across presentations.

Another approach that can be used in combination with or instead of any approach (FiFo, partial profiling, and HII) with moderately large numbers of attributes is an attribute-level overlap design. This means that, in each choice set, several attributes have the same level across all alternatives. This makes the choice task easier for respondents. With different attributes being “overlapped” in different choice sets, this ensures that attention is paid to all attributes instead of a limited set that respondents decided to focus on.^26^

FiFo has some limitations as well. First, it requires that all attributes within a domain have the same set of levels. If this is not possible, they cannot be combined in FiFo domains.

A second limitation is that the extended analysis is currently not facilitated by widely available software, such as Nlogit, SAS, or Stata. Specific programming was required to build the regression models that included additional parameters. Third, the same is true for creating the fractional factorial design. We developed 4 separate designs in Ngene and combined these into blocks of choice tasks. The overall design would have been more efficient if it had been produced in 1 step. This would have required additional programming in different software.

The main limitation of this study is that we did not directly compare the performance of FiFo to that of HII and partial profiling. It would not have been possible in the context of the current study, because no overarching constructs could be formed for the ABC Index. The comparison could be the focus of further research.

In conclusion, using a combination of folded-in and folded-out attributes seems to be a feasible approach for conducting a DCE with a high number of attributes. The analysis requires statistical models with additional parameters to address the increased complexity and to prevent the potential underestimation of the weights for folded-in attributes.

## Supplemental Material

9_Appendix.rjf_online_supp – Supplemental material for The Fold-in, Fold-out Design for DCE Choice Tasks: Application to Burden of Disease Click here for additional data file. Supplemental material, 9_Appendix.rjf_online_supp for The Fold-in, Fold-out Design for DCE Choice Tasks: Application to Burden of Disease by Lucas M. A. Goossens, Marcel F. Jonker, Maureen P. M. H. Rutten-van Mölken, Melinde R. S. Boland, Annerika H. M. Slok, Philippe L. Salomé, Onno C. P. van Schayck, Johannes C. C. M. in ‘t Veen, Elly A. Stolk and Bas Donkers in Medical Decision Making
