# Supplementary material for: The Fold-in, Fold-out Design for DCE Choice Tasks: Application to Burden of Disease
Source: Med Decis Making. 2019 May 29;39(4):450–60. doi: 10.1177/0272989X19849461 (PMC6613173; doi:10.1177/0272989X19849461)
Supplement: 9_Appendix.rjf_online_supp – Supplemental material for The Fold-in, Fold-out Design for DCE Choice Tasks: Application to Burden of Disease [file 9_Appendix.rjf_online_supp.docx]

# Appendix

This appendix contains the following items:

Figure A1: The Assessment of Burden of COPD questionnaire

Figure A2: Example of a choice set with a partial profiling design*

Figure A3: Example of a choice set with a hierarchical information integration design*

The OpenBUGS code that was used to fit the regression models

# **Figure A1** The Assessment of Burden of COPD questionnaire

| **On average, during the past week, how often did you feel:** | | | | | | | | | |
| --- | --- | --- | --- | --- | --- | --- | --- | --- | --- |
|  | Never | | | Hardly ever | A few times | Several times | Many times | A great many times | Almost all the time |
| **1** Short of breath at rest? | ☐ | | | ☐ | ☐ | ☐ | ☐ | ☐ | ☐ |
| **2**  Short of breath doing physical activities? | ☐ | | | ☐ | ☐ | ☐ | ☐ | ☐ | ☐ |
| **3** Concerned about getting a cold or your   breathing getting worse? | ☐ | | | ☐ | ☐ | ☐ | ☐ | ☐ | ☐ |
| **4** Depressed (down) because of your breathing   problems? | ☐ | | | ☐ | ☐ | ☐ | ☐ | ☐ | ☐ |
| **In general, during the past week, how much of the time:** | | | | | | | | | |
|  | | Never | | Hardly ever | A few times | Several times | Many times | A great many times | Almost all the time |
| **5** Did you cough? | | ☐ | | ☐ | ☐ | ☐ | ☐ | ☐ | ☐ |
| **6** Did you produce phlegm? | | ☐ | | ☐ | ☐ | ☐ | ☐ | ☐ | ☐ |
| **On average, during the past week, how limited were you in these activities because of your breathing problems:** | | | | | | | | | |
|  | | | Not limited at all | Very slightly limited | Slightly limited | Moderately limited | Very limited | Extremely limited | Totally limited/ or unable to do |
| **7** Strenuous physical activities (such as climbing stairs, hurrying, doing sports)? | | | ☐ | ☐ | ☐ | ☐ | ☐ | ☐ | ☐ |
| **8** Moderate physical activities (such as walking,   house work, carrying things)? | | | ☐ | ☐ | ☐ | ☐ | ☐ | ☐ | ☐ |
| **9**  Daily activities at home (such as dressing,   washing yourself)? | | | ☐ | ☐ | ☐ | ☐ | ☐ | ☐ | ☐ |
| **10** Social activities (such as talking, being with children, visiting friends/relatives)? | | | ☐ | ☐ | ☐ | ☐ | ☐ | ☐ | ☐ |
| **How often in the past week did you suffer from:** | | | | | | | | | |
|  | | | Never | Hardly ever | A few times | Several times | Many times | A great many times | Almost all the time |
| **11** Worry? | | | ☐ | ☐ | ☐ | ☐ | ☐ | ☐ | ☐ |
| **12** Listlessness? | | | ☐ | ☐ | ☐ | ☐ | ☐ | ☐ | ☐ |
| **13** A tense feeling? | | | ☐ | ☐ | ☐ | ☐ | ☐ | ☐ | ☐ |
| **14** Fatigue? | | | ☐ | ☐ | ☐ | ☐ | ☐ | ☐ | ☐ |
|  | | | | | | | | | |

**Figure A2:** Example of a choice set with a partial profiling design*


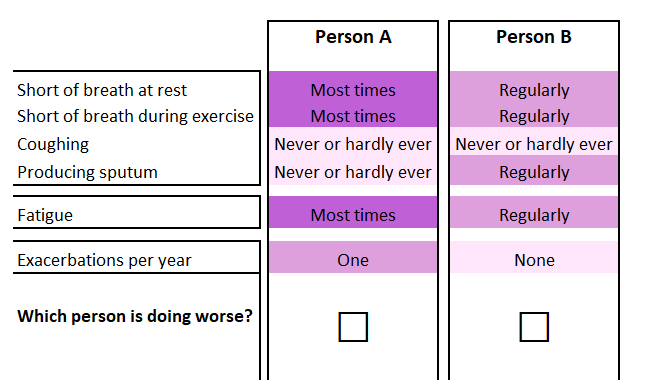


*In contrast to FiFo, not all attributes and dimensions are presented in each choice set. Note that the attributes from the Limitations and Mental Problems domains are not in this figure. Respondents are assumed to assume that the omitted attributes have the same level for both alternatives. Colour coding was added to facilitate comparison with the FiFo design. Darker shades indicate more severe problems.


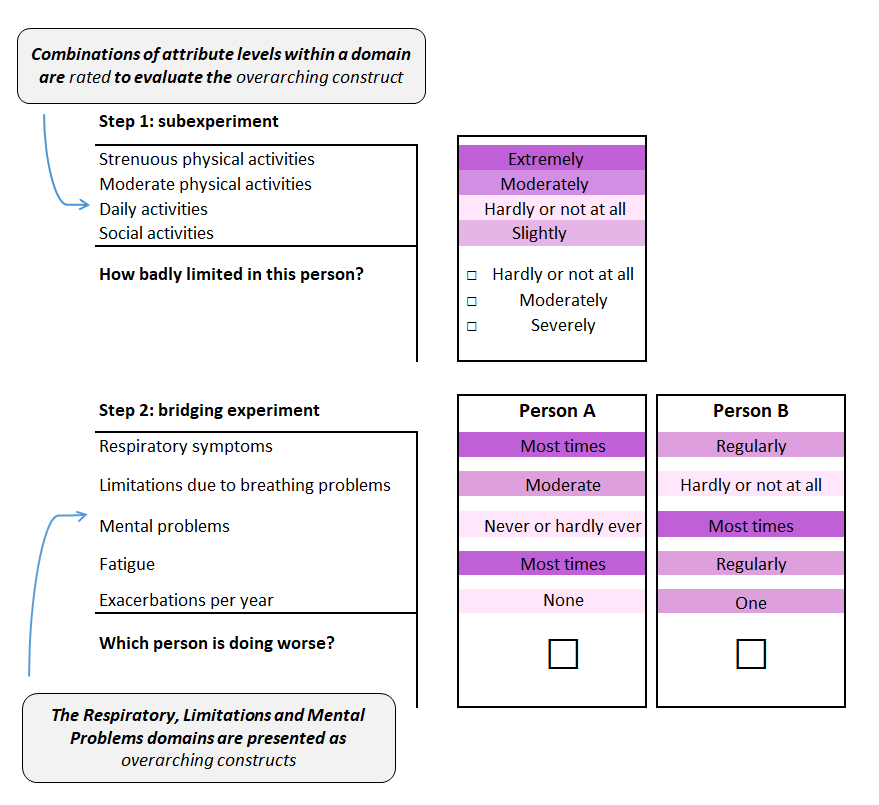
**Figure A3:** Example of a choice set with a hierarchical information integration design*

*In contrast to FiFo, not all attributes are presented in each choice set. Instead, attributes within a domain are valued in subexperiments, while overarching constructs are part of the bridging experiment. Colour coding was added to facilitate comparison with the FiFo design. Darker shades indicate more severe problems

**OpenBUGS code**

The following code was used in OpenBUGS for the Bayesian mixed logit model with additional scale parameters (1: complexity $\varphi$; 2-4: folding status of Symptoms, Limitations, Mental Problems domains λ):

model {

# likelihood

for (n in 1:N){

for (t in 1:T){

Y[n,t] <- 1

Y[n,t] ~ dcat2(phi[n,t]) }

for (t in 1:5){ phi[n,t] <- inprod( beta[n,], X[n,t,]) }

for (t in 6:T){ phi[n,t] <- (block1[n,t]+block2[n,t]+block3[n,t]+block4[n,t]) * (1+scale[1]) }

}

for (n in 1:N){

for (t in 6:T){

block1[n,t] <- beta[n,1] * (1 + A[n,t,1]*scale[2]) * X[n,t,1] +

beta[n,2] * (1 + A[n,t,2]*scale[2]) * X[n,t,2] +

beta[n,3] * (1 + A[n,t,3]*scale[2]) * X[n,t,3] +

beta[n,4] * (1 + A[n,t,4]*scale[2]) * X[n,t,4] +

beta[n,5] * (1 + A[n,t,5]*scale[2]) * X[n,t,5] +

beta[n,6] * (1 + A[n,t,6]*scale[2]) * X[n,t,6] +

beta[n,7] * (1 + A[n,t,7]*scale[2]) * X[n,t,7] +

beta[n,8] * (1 + A[n,t,8]*scale[2]) * X[n,t,8]

block2[n,t] <- beta[n,9] * (1 + B[n,t,9]*scale[3]) * X[n,t,9] +

beta[n,10] * (1 + B[n,t,10]*scale[3]) * X[n,t,10] +

beta[n,11] * (1 + B[n,t,11]*scale[3]) * X[n,t,11] +

beta[n,12] * (1 + B[n,t,12]*scale[3]) * X[n,t,12] +

beta[n,13] * (1 + B[n,t,13]*scale[3]) * X[n,t,13] +

beta[n,14] * (1 + B[n,t,14]*scale[3]) * X[n,t,14] +

beta[n,15] * (1 + B[n,t,15]*scale[3]) * X[n,t,15] +

beta[n,16] * (1 + B[n,t,16]*scale[3]) * X[n,t,16]

block3[n,t] <- beta[n,17] * (1 + C[n,t,17]*scale[4]) * X[n,t,17] +

beta[n,18] * (1 + C[n,t,18]*scale[4]) * X[n,t,18] +

beta[n,19] * (1 + C[n,t,19]*scale[4]) * X[n,t,19] +

beta[n,20] * (1 + C[n,t,20]*scale[4]) * X[n,t,20] +

beta[n,21] * (1 + C[n,t,21]*scale[4]) * X[n,t,21] +

beta[n,22] * (1 + C[n,t,22]*scale[4]) * X[n,t,22] +

beta[n,23] * (1 + C[n,t,23]*scale[4]) * X[n,t,23] +

beta[n,24] * (1 + C[n,t,24]*scale[4]) * X[n,t,24] +

beta[n,25] * (1 + C[n,t,25]*scale[4]) * X[n,t,25] +

beta[n,26] * (1 + C[n,t,26]*scale[4]) * X[n,t,26]

block4[n,t] <- beta[n,27] * X[n,t,27] +

beta[n,28] * X[n,t,28] +

beta[n,29] * X[n,t,29] +

beta[n,30] * X[n,t,30]

}}

# priors on scale parameters

for (s in 1:4){scale[s] ~ dnorm(0,0.01)}

# multivariate normal prior on beta

for (n in 1:N){ beta[n,1:V] ~ dmnorm(mu_beta[], Tau_beta[,]) }

mu_beta[1:V] ~ dmnorm(hyper_mu_beta[],hyper_tau_beta[,])

Tau_beta[1:V,1:V] ~ dwish(scale_beta[,],V)

for (b in 1:V){

hyper_mu_beta[b] <- 0

for (bb in 1:V){

hyper_tau_beta[b,bb] <- equals(b,bb)/100

scale_beta[b,bb] <- equals(b,bb)

}}

# Monitor SD of beta[n]

covar[1:V,1:V] <- inverse(Tau_beta[,])

for (b in 1:V){ SD[b] <- sqrt(covar[b,b]) }

# Log-likelihood for WAIC

for (n in 1:N){

for (t in 1:T){LL_task[n,t] <- -1 * log(1+ exp(phi[n,t])) }

LL_resp[n] <- sum(LL_task[n,])

}

LL <- sum(LL_resp[])

}}
